# Supplementary material for: New Carboxytriazolyl Amphiphilic Derivatives of Calix[4]arenes: Aggregation and Use in CuAAC Catalysis
Source: Int J Mol Sci. 2023 Nov 23;24(23):16663. doi: 10.3390/ijms242316663 (PMC10706699; doi:10.3390/ijms242316663)
Supplement: Supplementary file 1 [file ijms-24-16663-s001.zip › ijms-2718807-supplementary.pdf]

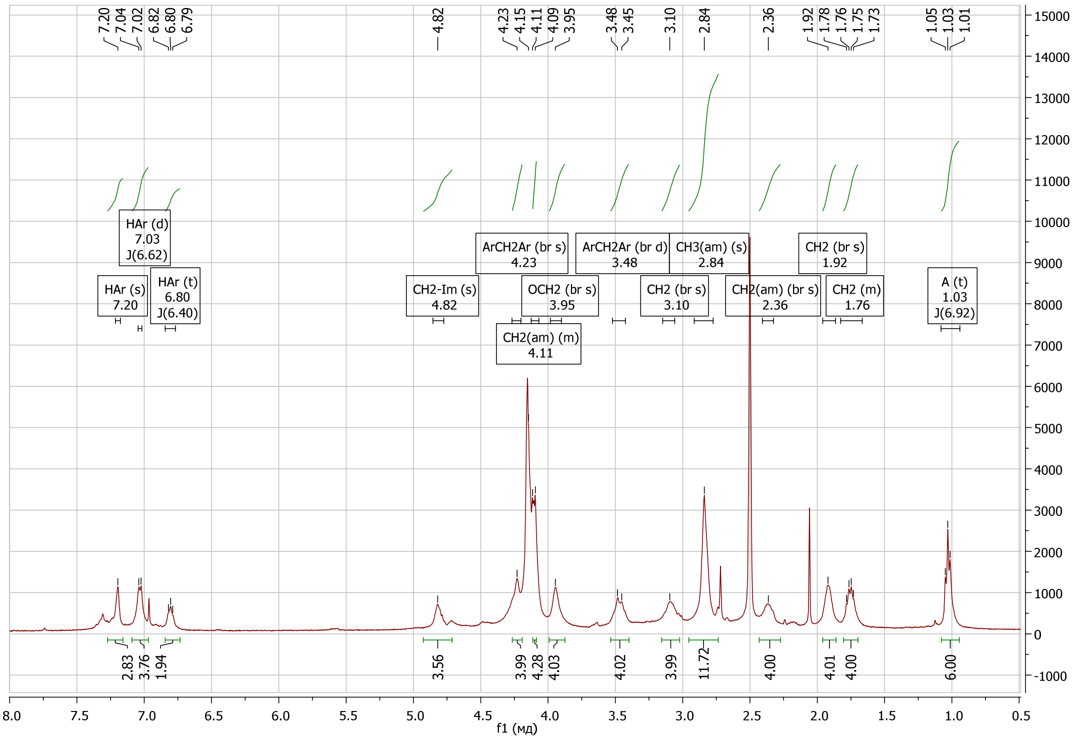

HAr (s)
7.20
HAr (d)
7.03
J(6.62)
HAr (t)
6.80
J(6.40)
CH2-Im (s)
4.82
ArCH2Ar (br s)
4.23
CH2(am) (m)
4.11
OCH2 (br s)
3.95
ArCH2Ar (br d)
3.48
CH2 (br s)
3.10
CH3(am) (s)
2.84
CH2(am) (br s)
2.36
CH2 (br s)
1.92
CH2 (m)
1.76
A (t)
1.03
J(6.92)
f1 (мд)

(a)

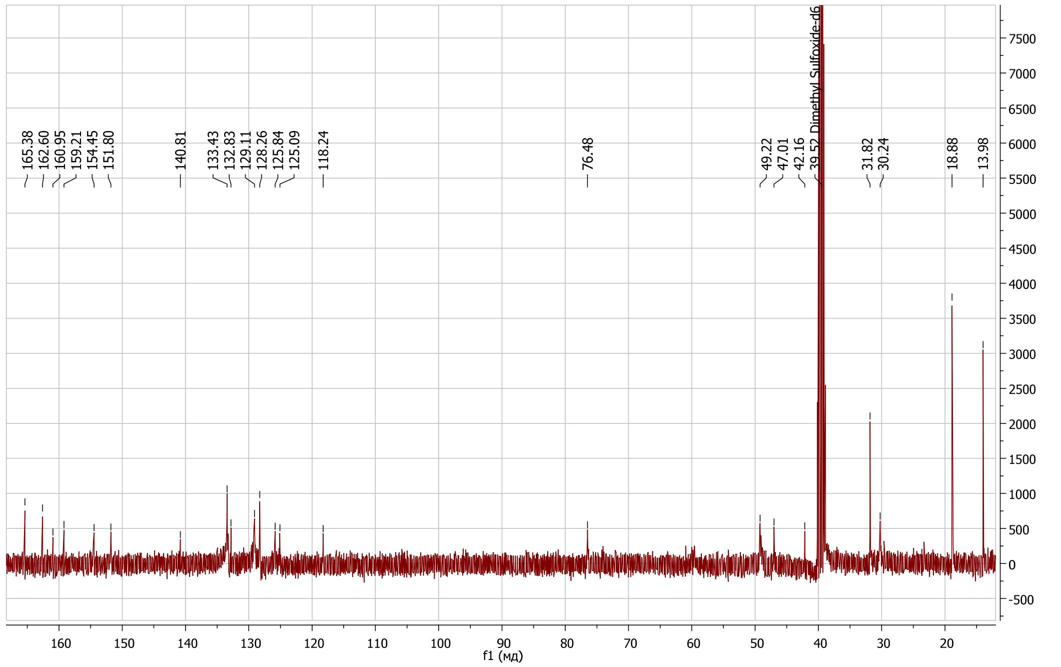

39.52 Dimethyl Sulfoxide-d6
f1 (мд)

(b)

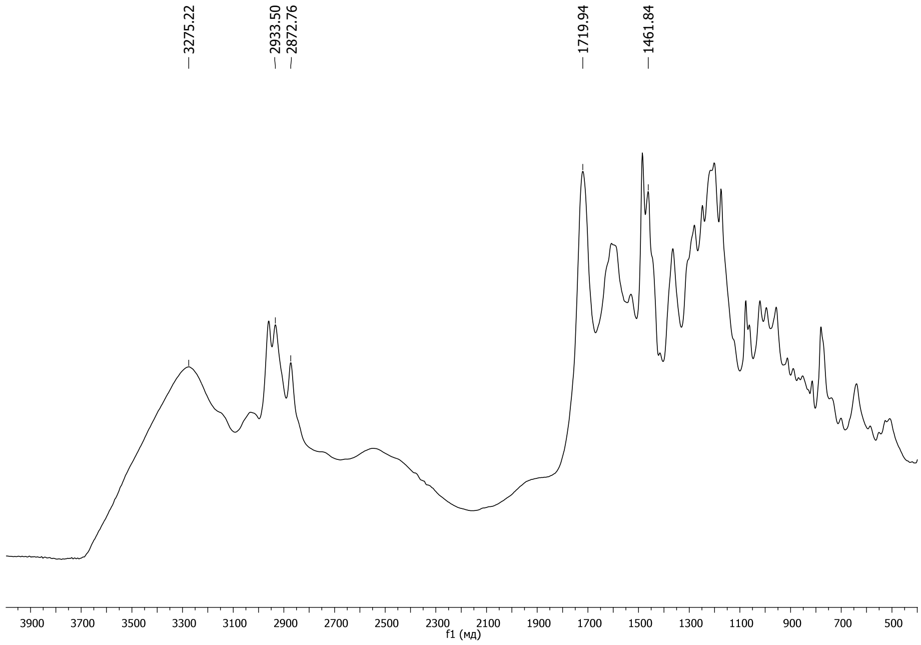
3275.22
2933.50
2872.76
1719.94
1461.84
3900
3700
3500
3300
3100
2900
2700
2500
2300
2100
1900
1700
1500
1300
1100
900
700
500
f1 (мд)

(c)

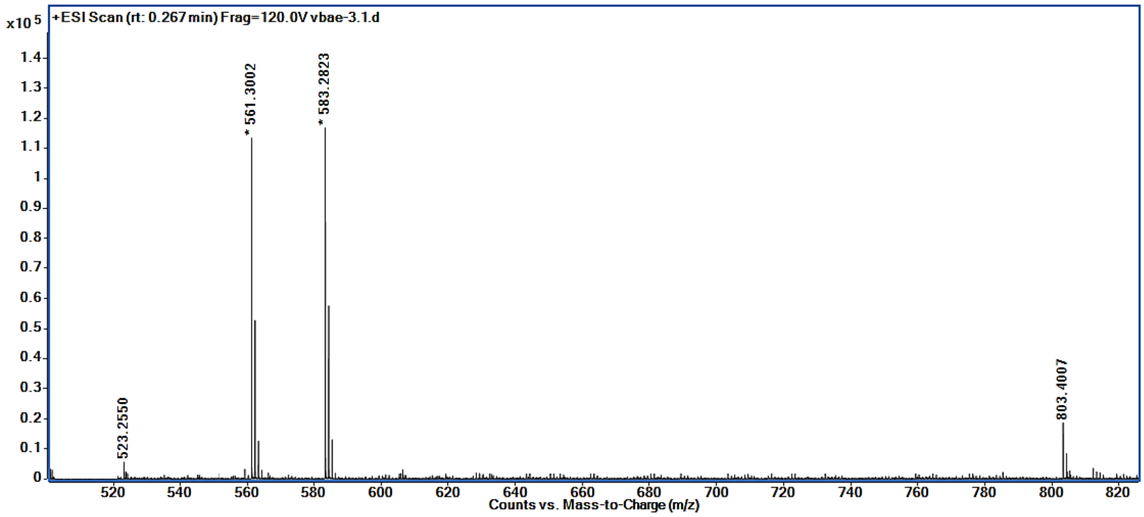
+ESI Scan (rt: 0.267 min) Frag=120.0V vbae-3.1.d
x10 5
* 561.3002
* 583.2823
523.2550
803.4007
Counts vs. Mass-to-Charge (m/z)

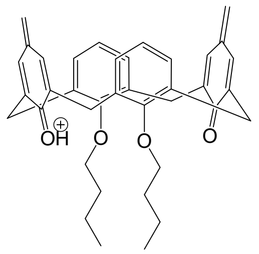

m/z: 561.3000 (100.0%), 562.3033 (41.1%), 563.3067 (8.2%), 564.3100 (1.1%)

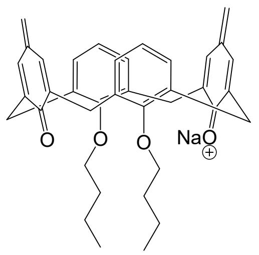

m/z: 583.2819 (100.0%), 584.2853 (41.1%), 585.2886 (8.2%), 586.2920 (1.1%)

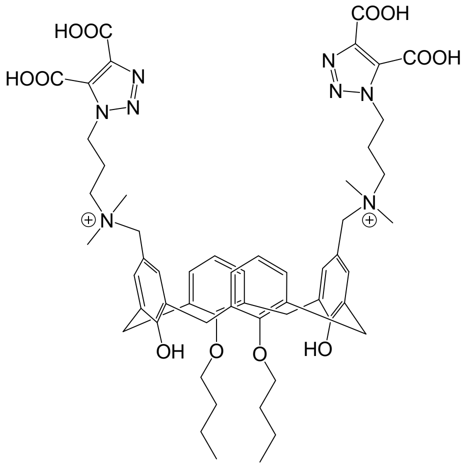

m/z: 523.2551 (100.0%), 523.7568 (60.6%), 524.2585 (18.0%), 524.7602 (3.5%), 523.7537 (3.0%), 524.2573 (2.5%), 524.2553 (1.8%), 524.7589 (1.5%)

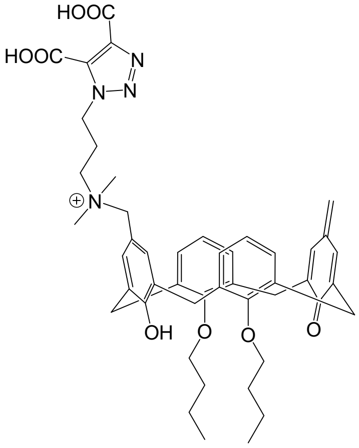

m/z: 803.4015 (100.0%), 804.4048 (50.8%), 805.4082 (12.6%), 806.4116 (2.1%), 805.4057 (1.6%), 804.3985 (1.5%)

(**d**)

Figure S1**.** NMR ^{1}H ((**a**), ^{13}C ((**b**), FT IR ((**c**) and HRESI MS ((**d**) spectra of 11,23-bis[(3-(4,5-dicarboxy-1,2,3-triazol-1-yl)propyl)dimethylammonium)methyl]-25,27-dihydroxy-26,28-dibutoxycalix[4]arene dichloride (**7**(**a**)**.**

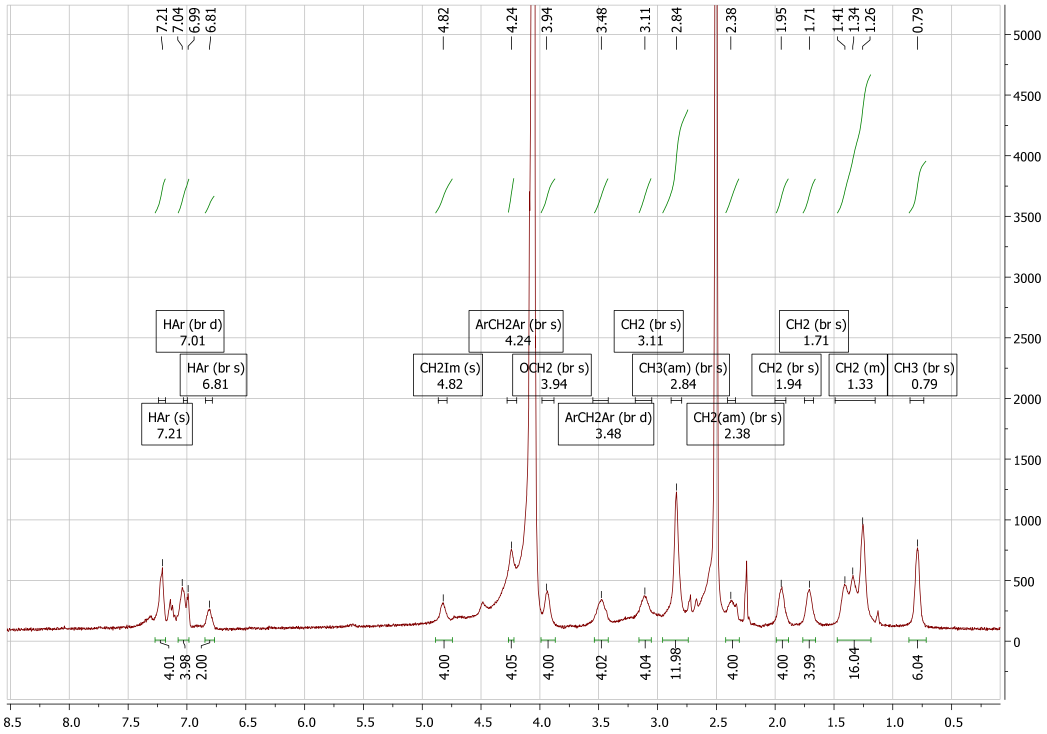
HAr (br d)
7.01
HAr (br s)
6.81
HAr (s)
7.21
CH2Im (s)
4.82
ArCH2Ar (br s)
4.24
OCH2 (br s)
3.94
ArCH2Ar (br d)
3.48
CH2 (br s)
3.11
CH3(am) (br s)
2.84
CH2(am) (br s)
2.38
CH2 (br s)
1.94
CH2 (br s)
1.71
CH2 (m)
1.33
CH3 (br s)
0.79

(a)

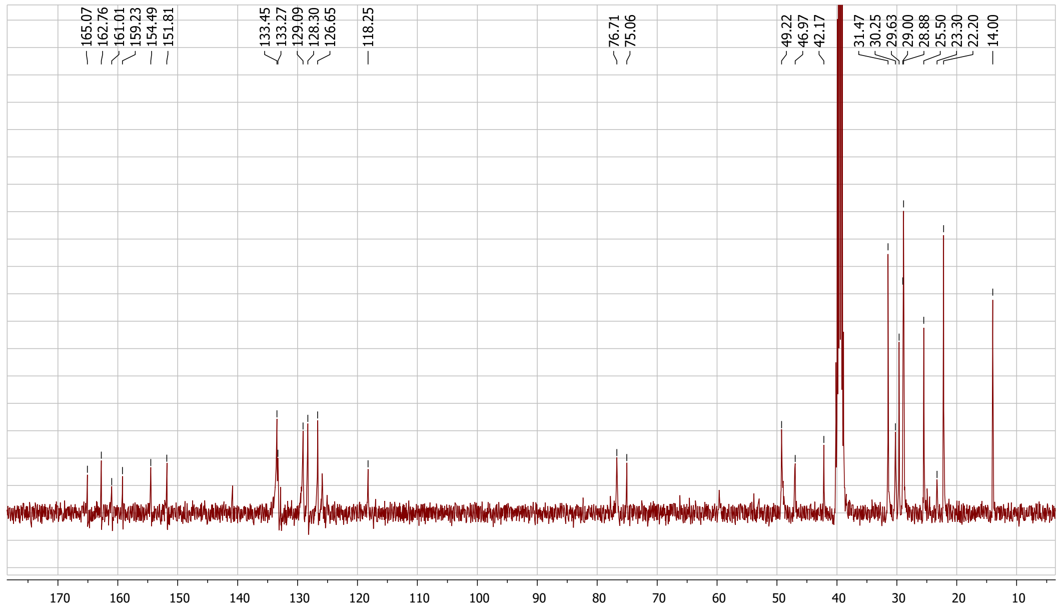

(b)

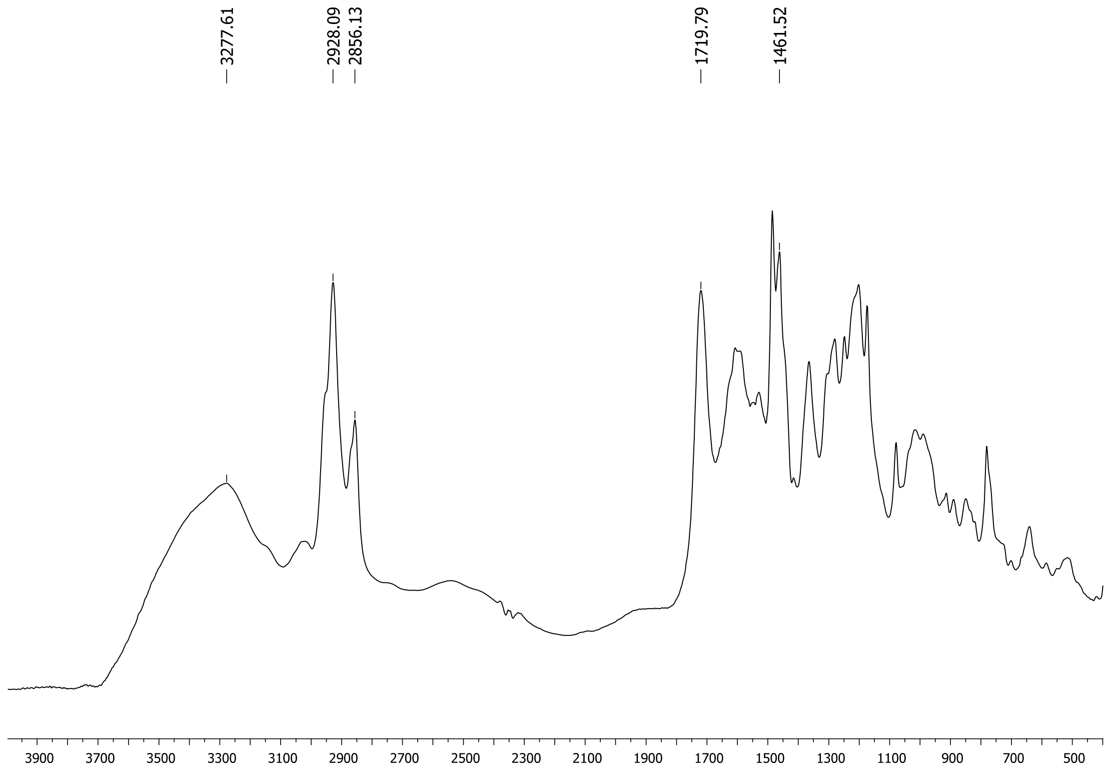
3277.61
2928.09
2856.13
1719.79
1461.52
3900
3700
3500
3300
3100
2900
2700
2500
2300
2100
1900
1700
1500
1300
1100
900
700
500

(c)

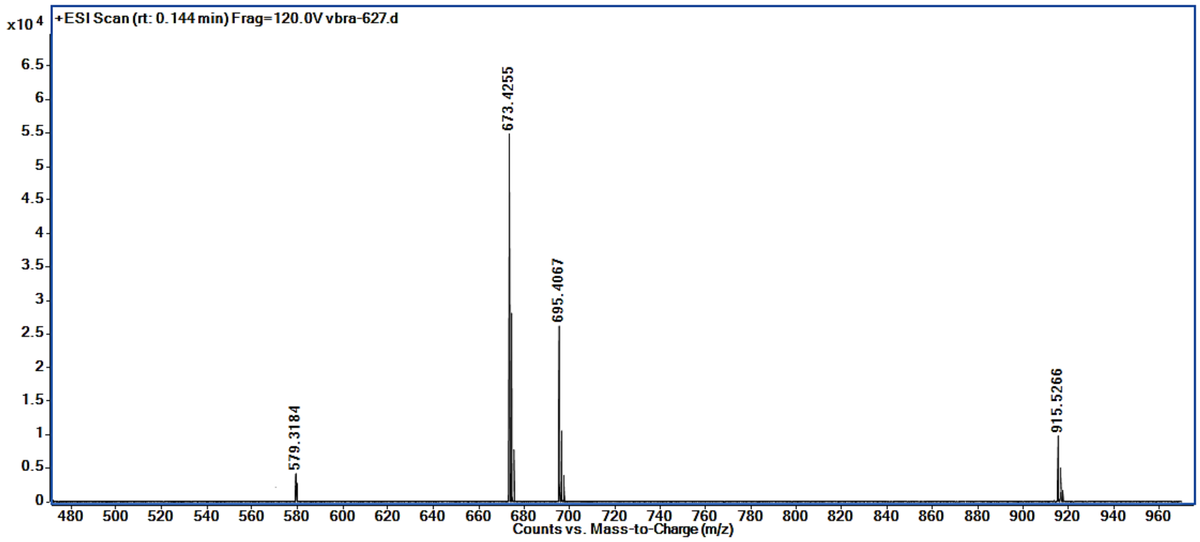
+ESI Scan (rt: 0.144 min) Frag=120.0V vbra-627.d
x10 4
673.4255
695.4067
915.5266
579.3184
Counts vs. Mass-to-Charge (m/z)

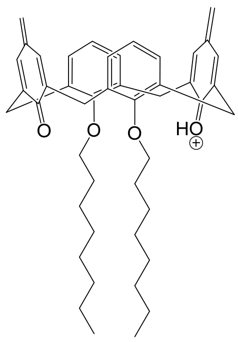

m/z: 673.4252 (100.0%), 674.4285 (49.8%),
675.4319 (12.1%), 676.4352 (1.9%)

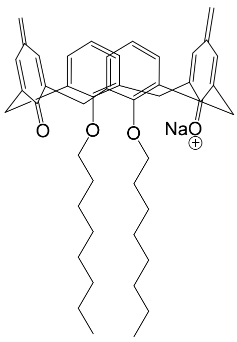

m/z: 695.4071 (100.0%), 696.4105 (49.8%),
697.4138 (12.1%), 698.4172 (1.9%)

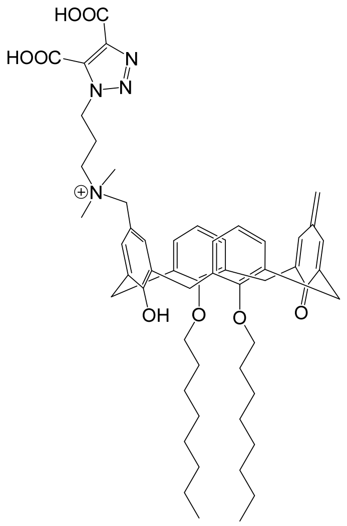

m/z: 915.5266 (100.0%), 916.5300 (59.5%),
917.5334 (17.4%), 918.5367 (2.5%),
917.5309 (1.6%), 916.5237 (1.5%)

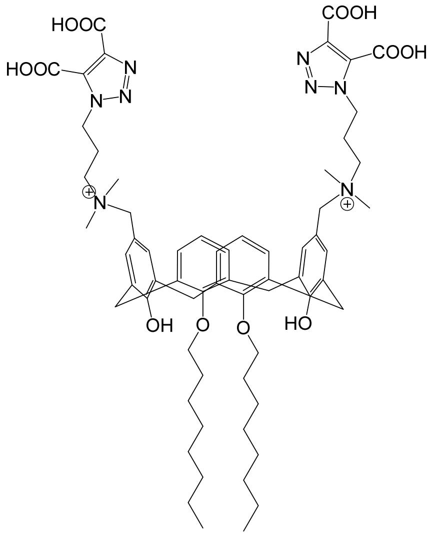

m/z: 579.3177 (100.0%), 579.8194 (69.2%),
580.3211 (23.6%), 580.8228 (5.3%),
579.8163 (3.0%), 580.3199 (2.5%),
580.3179 (2.0%), 580.8215 (1.7%)

(**d**)

Figure S2. NMR ^{1}H ((**a**), ^{13}C ((**b**), FT IR ((**c**) and HRESI MS ((**d**) spectra of 11,23-bis[(3-(4,5-dicarboxy-1,2,3-triazol-1-yl)propyl)dimethylammonium)methyl]-25,27-dihydroxy-26,28-dioctyloxycalix[4] arene dichloride (**7**(**b**).

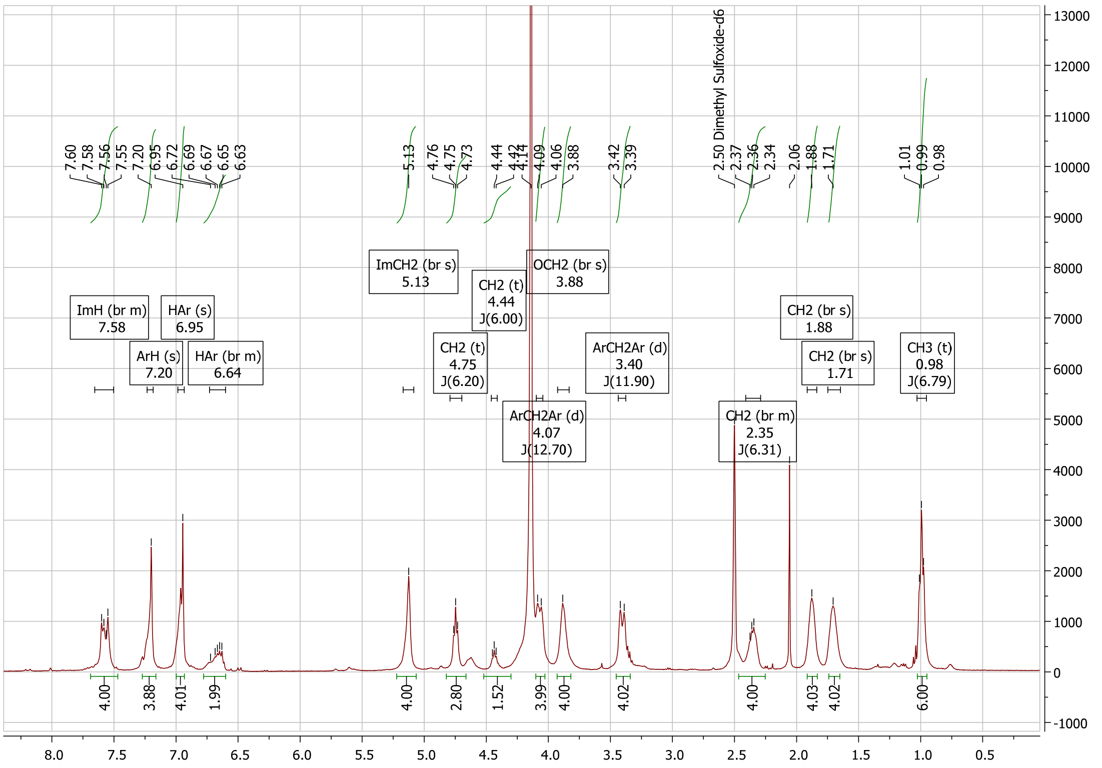

ImCH2 (br s) 5.13
CH2 (t) 4.44 J(6.00)
OCH2 (br s) 3.88
ImH (br m) 7.58
HAr (s) 6.95
CH2 (br s) 1.88
ArH (s) 7.20
HAr (br m) 6.64
CH2 (t) 4.75 J(6.20)
ArCH2Ar (d) 3.40 J(11.90)
CH2 (br s) 1.71
CH3 (t) 0.98 J(6.79)
ArCH2Ar (d) 4.07 J(12.70)
CH2 (br m) 2.35 J(6.31)
2.50 Dimethyl Sulfoxide-d6

(a)

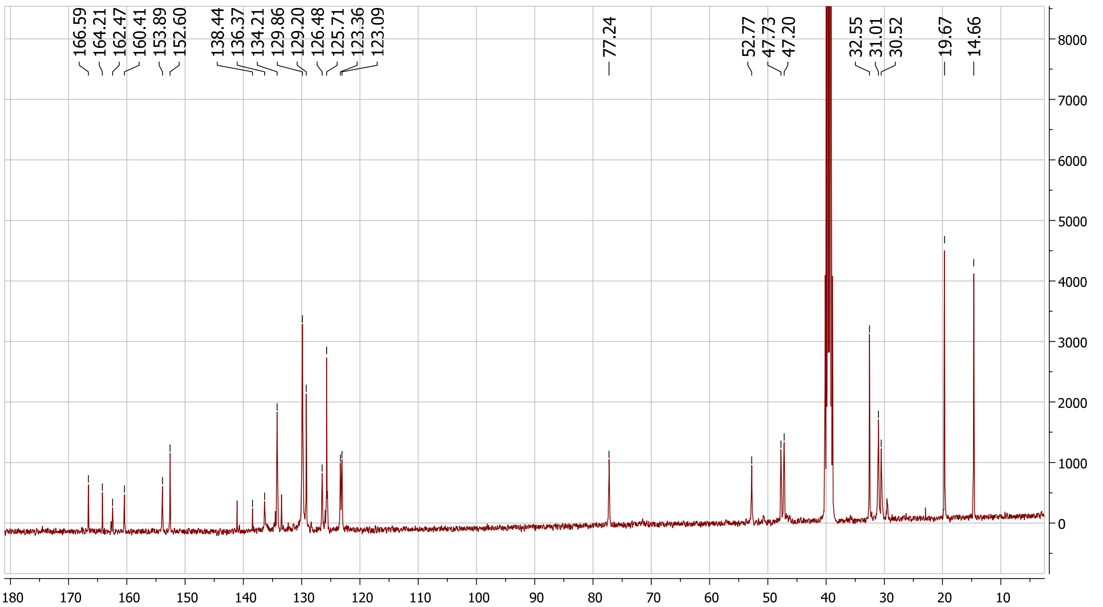

166.59
164.21
162.47
160.41
153.89
152.60
138.44
136.37
134.21
129.86
129.20
126.48
125.71
123.36
123.09
77.24
52.77
47.73
47.20
32.55
31.01
30.52
19.67
14.66

(b)

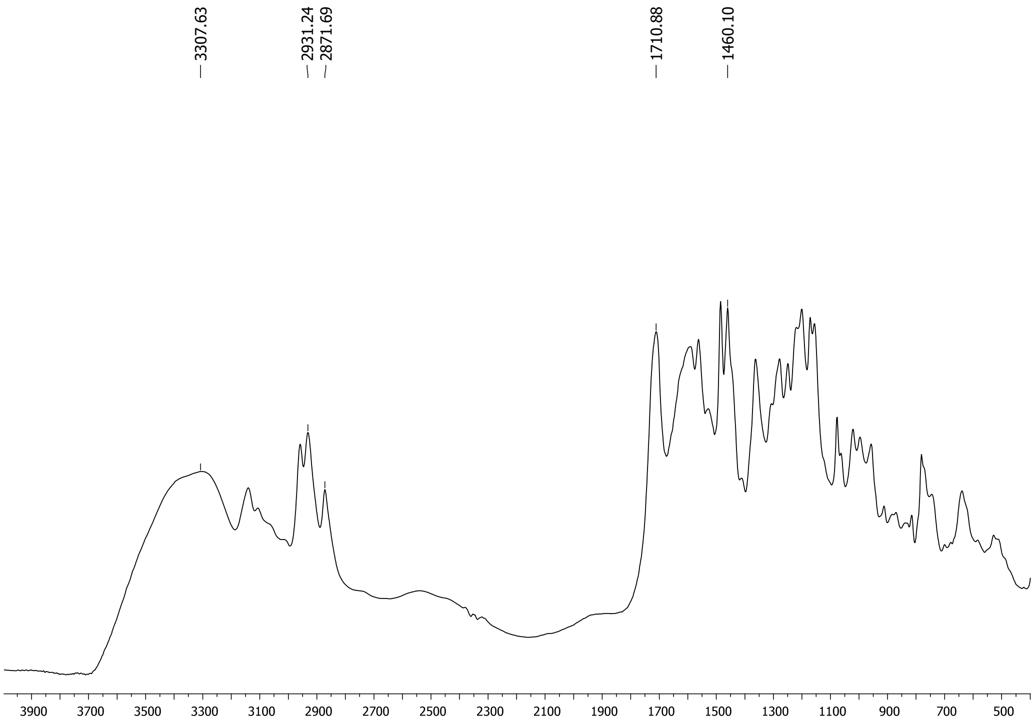
3307.63
2931.24
2871.69
1710.88
1460.10
3900 3700 3500 3300 3100 2900 2700 2500 2300 2100 1900 1700 1500 1300 1100 900 700 500

(c)

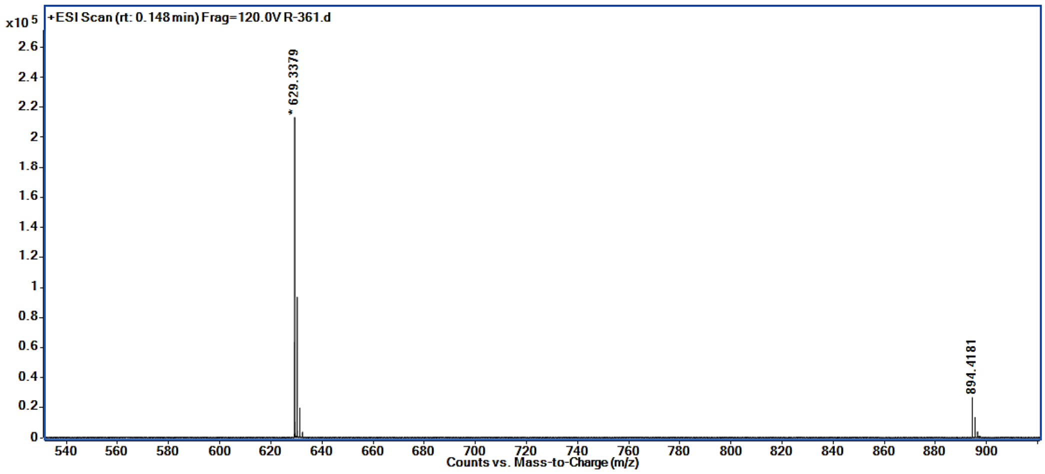
+ESI Scan (rt: 0.148 min) Frag=120.0V R-361.d
x10 5
* 629.3379
894.4181
Counts vs. Mass-to-Charge (m/z)

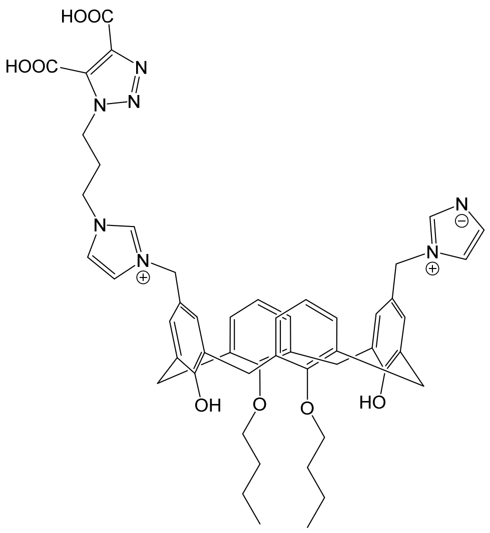

m/z: 894.4185 (100.0%), 895.4219 (55.2%), 896.4252 (14.9%), 897.4286 (2.6%), 895.4156 (2.6%), 896.4228 (1.6%), 896.4189 (1.4%)

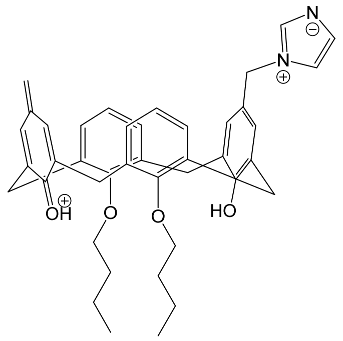

m/z: 629.3374 (100.0%), 630.3408 (44.3%), 631.3441 (9.6%), 632.3475 (1.3%)

(**d**)

Figure S3. NMR ^{1}H ((**a**), ^{13}C ((**b**), FT IR ((**c**) and HRESI MS ((**d**) spectra of 11,23-bis[(1-(3-(4,5-dicarboxy-1,2,3-triazol-1-yl)propyl))-1*H*-imidazolium)methyl]-25,27-dihydroxy-26,28-dibutoxycalix[4]arene dichloride (8(**a**).

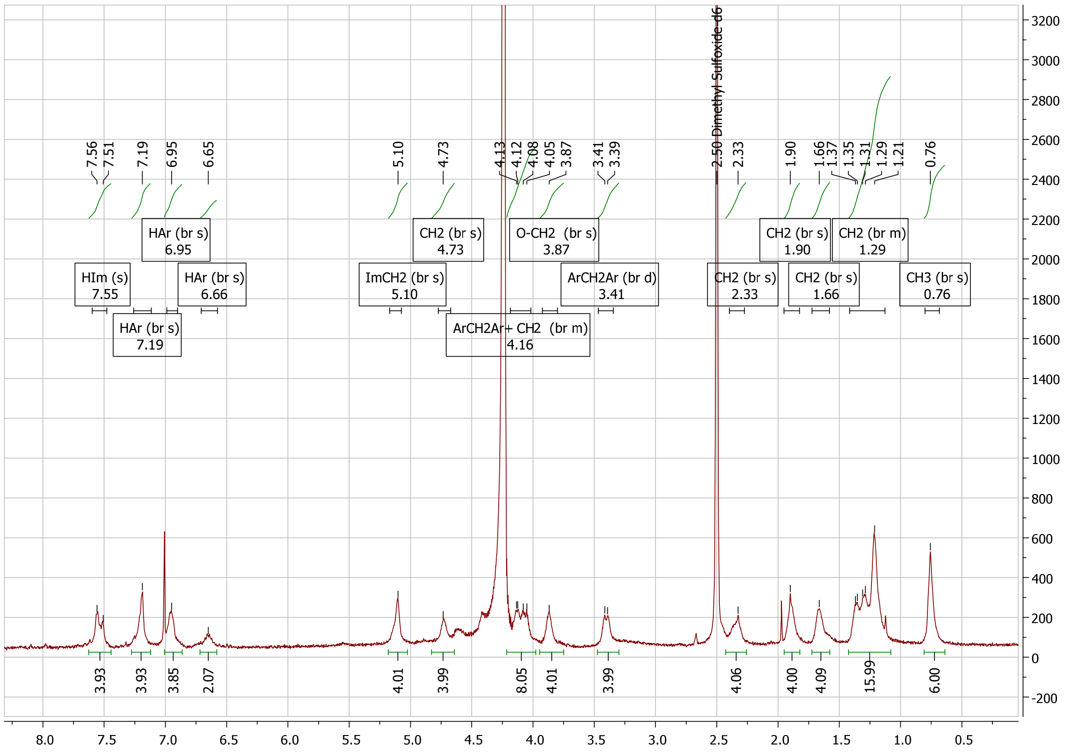

(**a**)

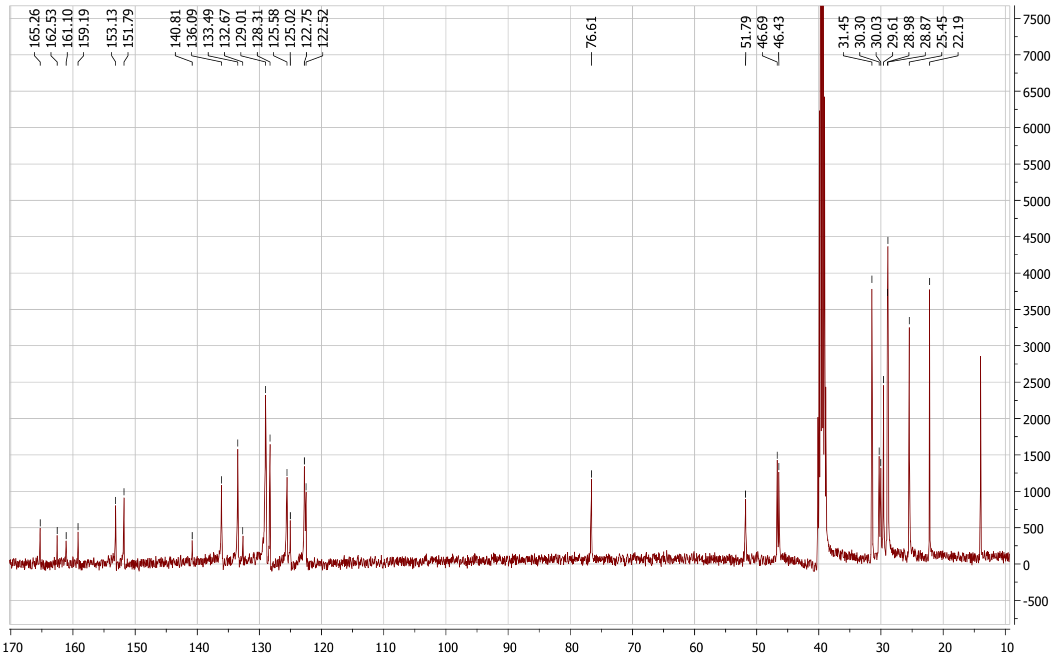
165.26
162.53
161.10
159.19
153.13
151.79
140.81
136.09
133.49
132.67
129.01
128.31
125.58
125.02
122.75
122.52
76.61
51.79
46.69
46.43
31.45
30.30
30.03
29.61
28.98
28.87
25.45
22.19
7500
7000
6500
6000
5500
5000
4500
4000
3500
3000
2500
2000
1500
1000
500
0
-500
170
160
150
140
130
120
110
100
90
80
70
60
50
40
30
20
10

(b)

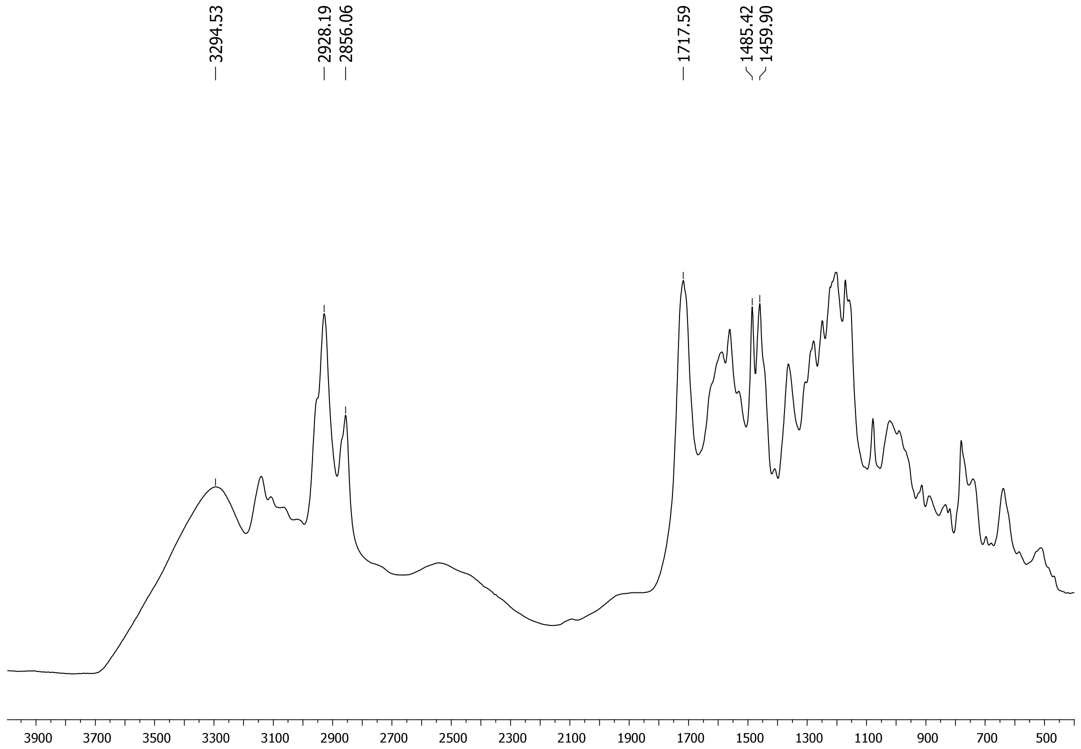
3294.53
2928.19
2856.06
1717.59
1485.42
1459.90
3900
3700
3500
3300
3100
2900
2700
2500
2300
2100
1900
1700
1500
1300
1100
900
700
500

(c)

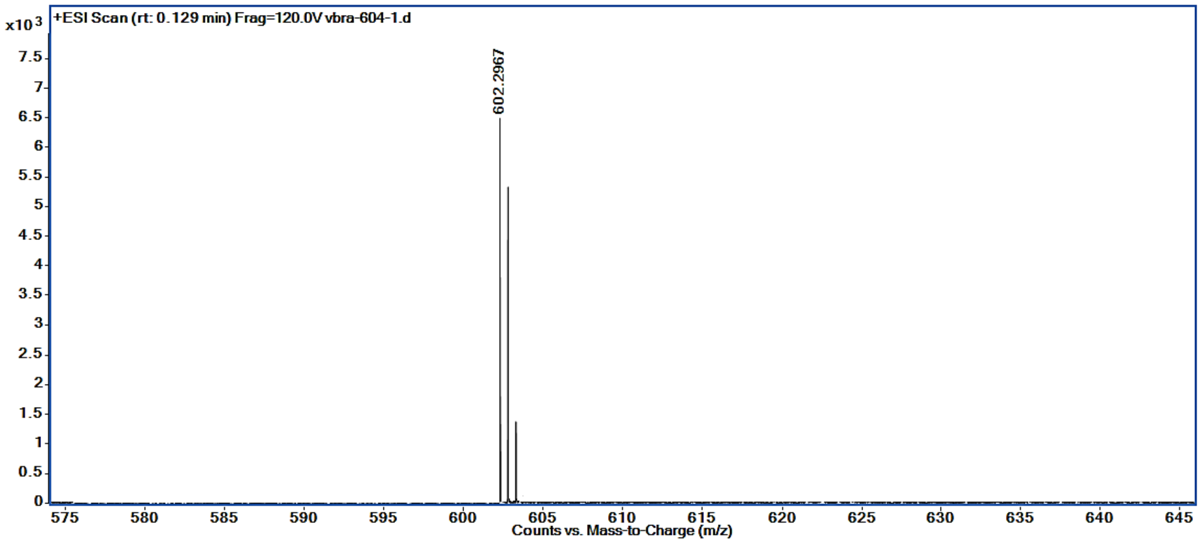

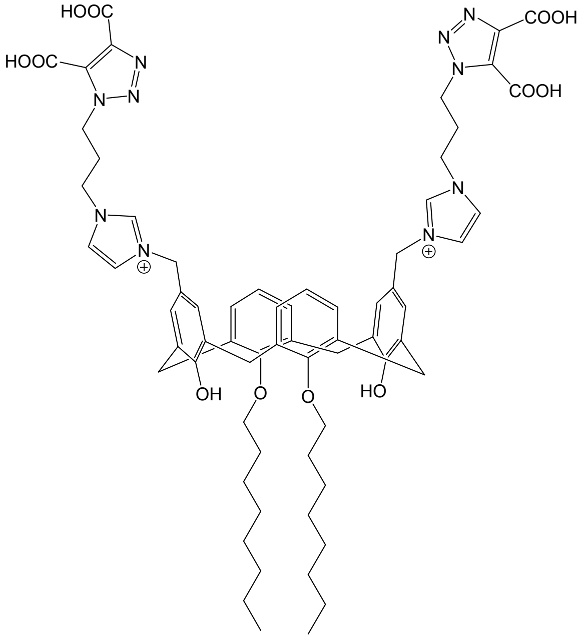

m/z: 602.2973 (100.0%), 602.7990 (71.4%),
603.3007 (25.1%), 603.8024 (5.8%),
602.7959 (3.7%), 603.2975 (2.6%),
603.2995 (2.5%), 603.8011 (1.8%)

(**d**)

Figure S4. NMR ^{1}H ((**a**), ^{13}C ((**b**), FT IR ((**c**) and HRESI MS ((**d**) spectra of 11,23-bis[(1-(3-(4,5-dicarboxy-1,2,3-triazol-1-yl)propyl))-1*H*-imidazolium)methyl]-25,27-dihydroxy-26,28 -dioctyloxycalix[4]arene dichloride (**8**(**b**)

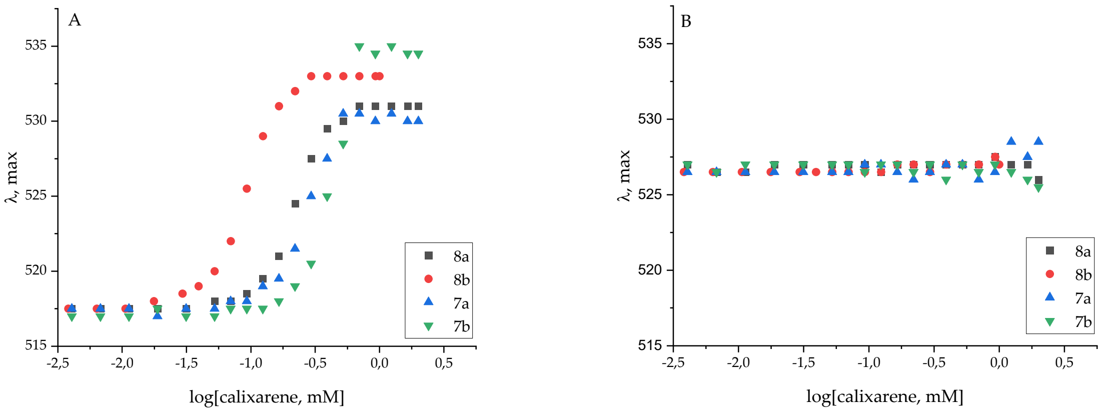

Figure S5. Dependence of the absorption maximum of Eosin Y ((**A**) and Rhodamine 6G ((**B**) on the concentration of calixarenes **7a-b** and **8a-b**. [dye] = 0.02 mM, [calixarene] = 0.001 – 2 mM, TRIS-HCl pH 7.4

Table S1. Dynamic light scattering (DLS) data aggregates formed by calixarenes **7a-b** and **8a-b**: Dh - average hydrodynamic particle diameter (nm), PDI - polydispersity index

Calixarene	Concentration, μM	Dh, nm	PDI
**8a**	1	667±47	0.522±0.019
	20	507±5	0.425±0.03
	70	587±23	0.406±0.04
	100	567±20	0.455±0.027
	200	571±45	0.422±0.098
**8b**	1	878±152	0.692±0.12
	20	427±80	0.573±0.018
	60	1072±213	0.843±0.126
	80	2446±183	1.000
	100	577.2±104	0.478±0.219
	200	1628±129	0.833±0.09
**7a**	1	653±65	0.537±0.051
	20	657±15	0.341±0.017
	40	934±40	0.308±0.039
	60	1091±6	0.247±0.013
	100	1393±69	0.184±0.038
**7b**	1	316±25	0.286±0.023

	20	310±7	0.219±0.012
	30	298±5	0.208±0.019
	40	303±9	0.174±0.018
	100	279±7	0.194±0.006

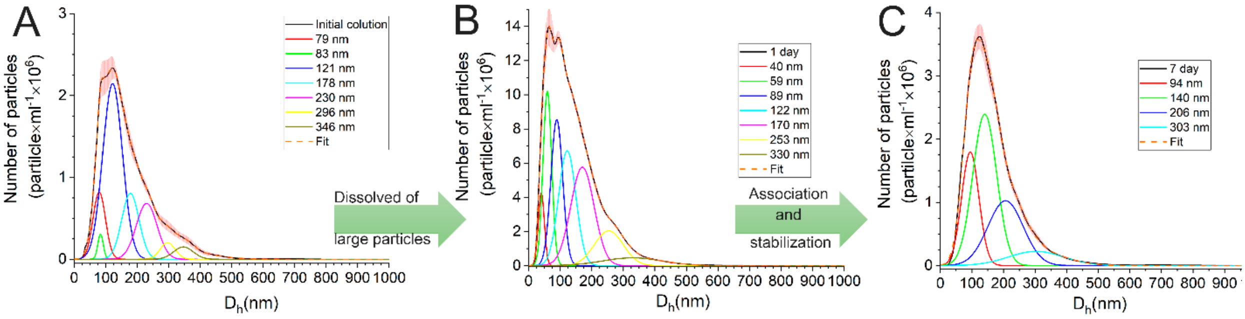

Figure S6. NTA analysis: (**A**) Initial solution of **7b** (10 minute after dissolution); (**B**) The same solution after 1 day; (**C**) The same solution after 7 day.

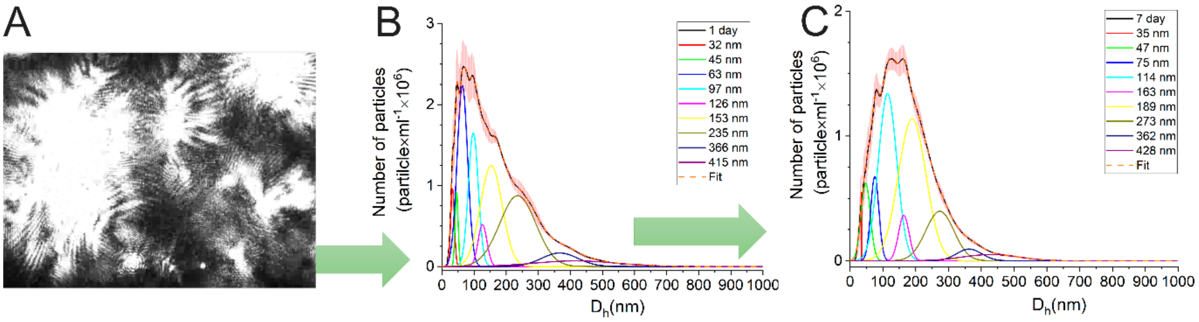

Figure S7. (**A**) Microphotography initial solution of **8a** by NTA (10 minute after dissolution); NTA analysis: (**B**) The same solution after 1 day; (**C**) The same solution after 7 day.

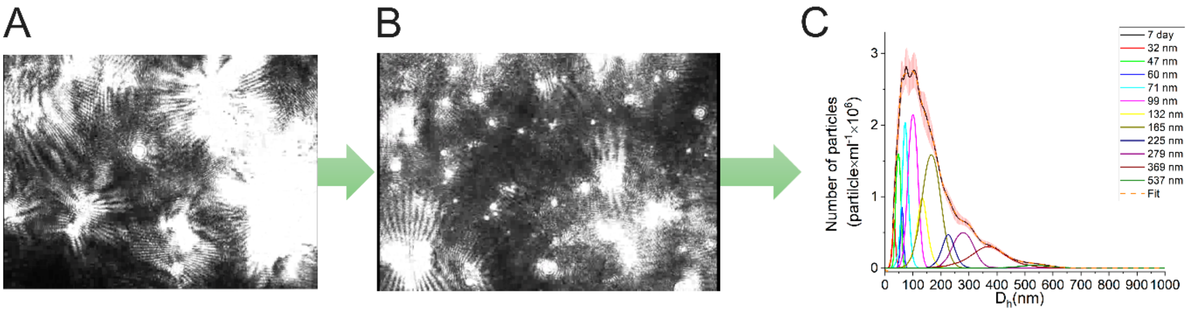

Figure S8. (**A**) Microphotography initial solution of **7a** by NTA (10 minute after dissolution);(**B**) The same solution after 1 day; (**C**) NTA analysis same solution after 7 day.
